# Evaluation of Multi-Layered Pancreatic Islets and Adipose-Derived Stem Cell Sheets Transplanted on Various Sites for Diabetes Treatment

**DOI:** 10.3390/cells9091999

**Published:** 2020-08-31

**Authors:** Yu Na Lee, Hye-Jin Yi, Yang Hee Kim, Song Lee, Jooyun Oh, Teruo Okano, In Kyong Shim, Song Cheol Kim

**Affiliations:** 1 Asan Institute for Life Sciences, Asan Medical Center, Seoul 05505, Korea; yuna426@mail.ulsan.ac.kr (Y.N.L.); 2hyejinyi@gmail.com (H.-J.Y.); kyh@amc.seoul.kr (Y.H.K.); gene77@amc.seoul.kr (S.L.); juyun1503@amc.seoul.kr (J.O.); 2 Department of Medical Science, Asan Medical Institute of Convergence Science and Technology (AMIST), Asan Medical Center, University of Ulsan College of Medicine, Seoul 05505, Korea; 3 Regenerative Medicine Research Center, Dalim Tissen Co., Ltd., 31, Yeonhui-ro, Mapo-gu, Seoul 03982, Korea; 4 Cell Sheet Tissue Engineering Center, Department of Pharmaceutics and Pharmaceutical Chemistry, University of Utah, Salt Lake City, UT 84112, USA; teruo.okano@utah.edu; 5 Department of Surgery & Department of Biomedical Engineering, AMIST, University of Ulsan College of Medicine &Asan Medical Center, Seoul 05505, Korea

**Keywords:** islet, transplantation, adipose-derived stem cell, multi-layered cell sheet, liver surface, peritoneal wall, subcutaneous site

## Abstract

Islet cell transplantation is considered an ideal treatment for insulin-deficient diabetes, but implantation sites are limited and show low graft survival. Cell sheet technology and adipose-derived stem cells (ADSCs) can be useful tools for improving islet cell transplantation outcomes since both can increase implantation efficacy and graft survival. Herein, the optimal transplantation site in diabetic mice was investigated using islets and stem cell sheets. We constructed multi-layered cell sheets using rat/human islets and human ADSCs. Cell sheets were fabricated using temperature-responsive culture dishes. Islet/ADSC sheet (AI sheet) group showed higher viability and glucose-stimulated insulin secretion than islet-only group. Compared to islet transplantation alone, subcutaneous AI sheet transplantation showed better blood glucose control and CD31+ vascular traits. Because of the adhesive properties of cell sheets, AI sheets were easily applied on liver and peritoneal surfaces. Liver or peritoneal surface grafts showed better glucose control, weight gain, and intraperitoneal glucose tolerance test (IPGTT) profiles than subcutaneous site grafts using both rat and human islets. Stem cell sheets increased the therapeutic efficacy of islets in vivo because mesenchymal stem cells enhance islet function and induce neovascularization around transplanted islets. The liver and peritoneal surface can be used more effectively than the subcutaneous site in future clinical applications.

## 1. Introduction

Diabetes mellitus occurs because of the loss or impaired function of insulin-secreting pancreatic beta cells. Type 1 diabetes (T1D) is characterized by beta cell destruction due to autoimmune defects. Compared to conventional insulin injections, recent therapeutic approaches aim to restore endogenous insulin production. Islet transplantation is the cell therapy option in practice for T1D management, with the potential to restore normal blood glucose regulation. Moreover, islet culture offers several advantages, including ensuring the quality of islet preparations [1] to possibly decreasing allograft tissue immunogenicity [2]. However, this approach is limited by requiring an adequate functional islet number to achieve normal glycemic levels. Additionally, the loss of functional islet mass has been associated with stable islet engraftment [3].

The liver has become the most widely used islet implantation site in clinical trials because it provides nutritional and physical support. However, the hepatic portal may be considered a hostile environment for infusion that may limit successful islet engraftment and function [4,5,6]. Therefore, many investigations have pursued alternative pancreatic islet implantation sites to optimize islet engraftment and function, reduce necessary implantation mass, and decrease immunogenicity [7]. Several extrahepatic transplant sites have been explored to avoid intrahepatic complications, including kidney capsule, peritoneum, omental pouch, spleen, and subcutaneous sites [8,9,10,11], but they have limitations to show clinical effectiveness or significant advantages over the hepatic site [12].

Cell sheet technology was developed based on a novel technique for culturing and harvesting cells using temperature-responsive culture dishes first reported in 1990. The hydrophilic and hydrophobic properties of the temperature-sensitive material poly (N-isopropylacrylamide) (PIPAAm) could be altered through temperature changes, resulting in control over cell attachment. Cell sheet technology can be used to harvest cells without utilizing proteolytic enzymes. Thus, the cell-cell junctions, extracellular matrix, and adhesion proteins are effectively preserved, allowing the constructed tissue to have a high cell density and uniform cell distribution, and mimic the native tissue more closely [13,14]. This approach has numerous advantages over conventional tissue engineering techniques, including cell injection and cell-seeded scaffold transplantation. Cell sheet technology has been tested as a potential approach for pancreatic islet transplantation to explore alternative transplantation sites. In the previous study, Yamato et al. constructed islet cell sheets and compared transplantation site between subcutaneous space and liver surface. On the liver surface, relatively small mass of islet cell sheet was needed to achieve normoglycemia than that at subcutaneous site [15]. However, in this study, the intact islets were dissociated into a single cell, which may have limitations in actual clinical application. It is known that the tertiary spheroidal structure of islets is important for the normal physiological function of islets [16,17]. Therefore, it is necessary to develop a new cell sheet technique that would be applicable to various sites while maintaining this intact islet structure. In this study, we used stem cell sheet as a tool for delivery of intact islets and simultaneously compared transplantation sites, subcutaneous site, liver surface, peritoneal wall, which are clinically applicable transplant sites.

Because mesenchymal stem cells (MSCs) exert a protective role toward islets, previous studies have demonstrated that cell sheets comprising islets and MSCs enhanced islet engraftment and function [18,19,20,21]; among these studies, most were performed in subcutaneous tissues. A subsequent study confirmed that a suitable supporting cell type was tested to construct cell sheets with islets, and favorable results were observed with MSCs as a supporting source for islet cell sheets [22].

Subcutaneous spaces offer the best surgical advantages regarding implantation accessibility, graft removal, and routine biopsy monitoring with minimal complications [23]. However, the clinical application of this site is hampered by low oxygen tension due to poor perfusion, consequently resulting in the need to transplant comparatively high amounts of viable islets to control diabetes [24,25]. For islet cell sheets, suitable transplantation sites have not been comparatively examined, and the islet number required for transplantation is undetermined. Here, we evaluate the impact of subcutaneous islet/ADSC sheet (AI sheet) on islet engraftment and function in three extrahepatic sites: the liver surface, peritoneal wall, and subcutaneous site. The ADSC sheet facilitates minimally invasive and facile cell delivery to extrahepatic sites and enhances islet survival. Furthermore, this cell sheet strategy enables the restoration of euglycemia with a minimal mass of islets and may be a feasible and useful modality for the cell-based therapy of diabetes mellitus.

## 2. Materials and Methods

### 2.1. Experimental Animals

Male Sprague-Dawley rats (8 weeks, 200–250 g) and BALB/c-nu mice (8 weeks, 20–25 g) purchased from Orient Bio (Gyeonggi-do, Korea) were used as donors and recipients, respectively. In our study, rats were selected as the animal donors because they provide a higher yield of islet. In addition, we used congenitally athymic nude mice as recipients to avoid any interference of immune rejection on the outcome of results.

### 2.2. Rat Islet Isolation

Rat islets were isolated and purified using the Ficoll purification method. Briefly, the pancreas of each rat was distended with a 10-mL intraductal injection of Hank’s balanced salt solution (HBSS) containing collagenase type XI (800 U/mL, Sigma-Aldrich, St. Louis, MO, USA). Pancreatic tissue was surgically removed, and incubated in a water bath at 37 °C. After 26 min of incubation, digestion kinetics was rapidly slowed down by addition of cold HBSS supplemented with 20% fetal bovine serum (FBS, Gibco, Invitrogen, Carlsbad, CA, USA). Digested pancreatic tissue was mechanically disrupted by filtrating through a mesh (400 μm pore size) and washed with cold HBSS supplemented with 5% FBS. Islets were purified by discontinuous density gradient centrifugation using Ficoll (Sigma-Aldrich, St. Louis, MO, USA). Islet numbers and purity were determined via dithizone staining. Before experiments, islets were cultured overnight in RPMI 1640 medium (11.11 mM glucose, Gibco) supplemented with 10% FBS.

### 2.3. Human Islet and hADSC Isolation

Pancreatic tissue collected from a partial pancreatectomy due to benign pancreatic disease was used for islet isolation after obtaining informed consent from patients (25-year-old male and 57-year-old male). Human islet use was approved by the Ethics Committee of Asan Medical Center (IRB: 2019-0442). Islets were isolated by the Ricordi method [26] using Liberase MTF (Roche, Indianapolis, IN, USA) and purified on a continuous density gradient of iodixanol (Optiprep; Nycomed Pharma AS, Oslo, Norway) using the Cobe 2991 cell separator (Gambro, Lakewood, CO, USA). Islets were cultured for 24 h in CMRL-1066 medium (Mediatech, Herndon, VA, USA) before transplantation. Human adipose tissue was obtained from donors receiving abdominal surgery at Asan Medical Center (55-year-old male, 33-year-old female, and 40-year-old female). The study was approved by the Asan Medical Center Institutional Review Board (IRB: 2012-0244), and all donors provided informed consent. The hADSCs were isolated from adipose tissue using collagenase type I and cultured in low-glucose Dulbecco’s modified Eagle’s medium (Gibco, Invitrogen), as previously described (Zuk et al., 2002). The cells were classified as hADSCs based on adherence to plastic and expression of the surface markers CD90, CD29, CD73, CD 31, CD45 and MHC I (BD Pharmingen, San Diego, CA, USA). All experiments were performed using hADSCs after 3–5 passages (Supplementary Figure S1).

### 2.4. Cell Sheet Fabrication Using Rat or Human Islets with hADSCs

The hADSCs were cultured on temperature-responsive dishes (35 mm UpCell^TM^ dishes; Thermo Fisher Scientific, MA, USA) to form hADSC sheets. The polymer dishes are hydrophobic at 37 °C; at 20 °C, the dishes become hydrophilic and detach from cell sheets, which maintain intact cell-cell junctions. The hADSCs were seeded on temperature-sensitive dishes and cultured until confluence. Isolated islets were transferred onto confluent hADSC dishes and cultured for 24 h to induce islet attachment to hADSCs. The UpCell dishes were maintained at 37 °C in a lower-temperature incubator until experimentation. The cell sheets were detached by lowering the temperature at 20 °C to harvest multi-layered AI sheets.

### 2.5. Live/Dead Cell Assay

Islet viability was assessed using a LIVE/DEAD viability/cytotoxicity kit (ThermoFisher Scientific, Middletown, NJ, USA). Briefly, islets or AI sheets were placed in 35-mm Petri dishes. EthD-1 and calcein AM solution in Dulbecco’s phosphate-buffered saline (DPBS, Gibco, Invitrogen) were added to the sample at final concentrations of 4 and 2 μM, respectively. After 30 min of incubation at room temperature, the sample was subjected to fluorescence microscopy. Dead cells were stained red; viable cells were stained green.

### 2.6. Glucose-Stimulated Insulin Secretion Assay

Prior to glucose stimulation to the islets, any residual insulin released from the islets was removed by washing with Krebs-Ringer buffer and preincubated for 1 h in Krebs-Ringer buffer before the experiment. Islets were stimulated with low (3.3 mM) or high (16.7 mM) glucose for 1 h in shaking incubator. The sample supernatants were collected, and the released insulin concentration was analyzed using ELISA (Mercodia, Uppsala, Sweden). The stimulation index (SI) was defined as the ratio of insulin levels at 16.7 mM glucose to that at 3.3 mM glucose.

### 2.7. Islet and Islet/hADSC Sheet Transplantation

Diabetes was induced in BALB/c-nu mice by a single intraperitoneal injection of streptozotocin (250 mg/kg, Sigma-Aldrich, St. Louis, MO, USA). Diabetic mice with blood glucose >300 mg/dL for more than 2 consecutive days (Accu-Check, Roche, Indianapolis, IN, USA) were used as recipients. Islets or AI sheets were transplanted onto the liver surface, subcutaneous site, and peritoneal wall of diabetic mice. In addition, ADSC sheets without islets were transplanted onto the liver surface, subcutaneous site, and peritoneal wall of diabetic mice as a control. For liver and peritoneal surface transplantation, the surface of the recipient site was scratched with a dry gauze/cotton swab before sheet transplantation to induce adhesion [27]. Wounds were gently induced at the peritoneal wall to enhance cell sheet attachment. The surface roughness increased, but the transplantation was performed in a manner that severe bleeding or rupture was not observed. The AI sheet with the shifter membrane was transplanted on the subcutaneous site, liver surface, and peritoneal wall for 5 min. Thereafter, the shifter membrane was removed, leaving the AI sheet. Body weight was monitored daily to assess animal condition, activity, and blood glucose levels. Furthermore, we prepared rat islet insulinoma cell clusters stained with PKH26 Red fluorescent cell linker kit(Sigma-Aldrich, St. Louis, MO, USA), transplanted them into a sheet, and confirmed the survival of the transplanted cells via IVIS imaging at three sites to track the distribution of transplanted cells. Fluorescence images were processed using Living Image V.3.2 (Caliper Life Science, Inc., Hopkinton, MA, USA).

### 2.8. Intraperitoneal Glucose Tolerance Test

One month after islet transplantation, recipient mice were subjected to an intraperitoneal glucose tolerance test (IPGTT) to evaluate islet graft function. Diabetic and normal mice were used as controls. After 8 h of fasting, the mice received a glucose solution intraperitoneally (2 g/kg). Blood glucose levels were determined at 0, 5, 15, 30, 60, and 120 min after glucose administration. The area under the curve (AUC) of the glucose levels was calculated for each mouse.

### 2.9. Histological Analysis

To acquire frozen tissue blocks, resected tissues were fixed in 4% paraformaldehyde solution for 2 h, embedded in Tissue-Tek (Sakura Finetek, Torrance, CA, USA), and sectioned (6 μm). To identify rat insulinoma and ADSC distribution in grafts, rat insulinoma and ADSCs were labeled with a fluorescent cell linker (rat insulinoma; PKH 26 and ADSC; PKH 67, Sigma-Aldrich, St. Louis, MO, USA) following the manufacturer’s protocol before sheet fabrication. For immunofluorescence staining, sections were incubated overnight at 4 °C with primary rabbit monoclonal antibodies against insulin (1:1000; Abcam, Cambridge, MA, USA) for islets, or primary rabbit polyclonal antibodies against CD31 (1:100; Abcam) for endothelial cells. After washing, the slides were treated with Alexa Fluor 555 or 488 anti-rabbit IgG antibodies (1:1000; Invitrogen) for 1 h at room temperature. Nuclei were counterstained with 4,6-diamidino-2-phenylindole dihydrochloride (DAPI; Dako, Glostrup, Denmark), and fluorescently imaged using fluorescence microscopy (EVOS; ThermoFisher Scientific, Middletown, NJ, USA). Immunohistochemistry of human islet transplanted tissues was performed using primary antibodies against insulin, CD31, and Ki67 (dilution 1:1000, 1:200, 1:200, respectively, Abcam, Cambridge, UK). Formalin-fixed, paraffin-embedded sections (4-μm thickness) were deparaffinized, dehydrated through a graded alcohol series, blocked with hydrogen peroxide, and dried for 10 min at room temperature followed by 20 min in a 65 °C incubator. An automated slide preparation system (Benchmark XT; Ventana Medical Systems Inc., Tucson, AZ, USA) with an OptiView DAB Detection Kit (Ventana Medical Systems) was used for immunohistochemistry. Hematoxylin-eosin (H&E) staining and immunohistochemical staining were performed.

### 2.10. Statistical Analysis

Data are presented as means ± standard deviation of the mean (SD). A paired 2-tailed *t*-test was applied to compare two groups; ANOVA with Tukey’s multiple comparison test was used for comparisons of more than two groups. The sample numbers for each experiment are described in the relevant figure legends. A *p*-value <0.05 indicated a statistically significant difference.

### 2.11. Ethics Approval and Consent to Participate

This animal study was reviewed and approved by the Institutional Animal Care and Use COMMITTEE (IACUC No. 2015-01-193) of Asan Institute for Life Sciences. The committee abides by the Institute of Laboratory Animal Resources (ILAR) guidelines. All experiment protocols of human ADSCs and islet isolation were carried out according to the guidelines and with the approval of the Institutional Review Board of Asan Medical Center (IRB number: 2012-02(ADSCs) and 2019-0442 (islets), Seoul, Republic of Korea). We obtained written informed consent from all patients who participated in this study.

## 3. Results

### 3.1. Preparation of Multilayered Islet and ADSC Sheets

**Figure 1:**
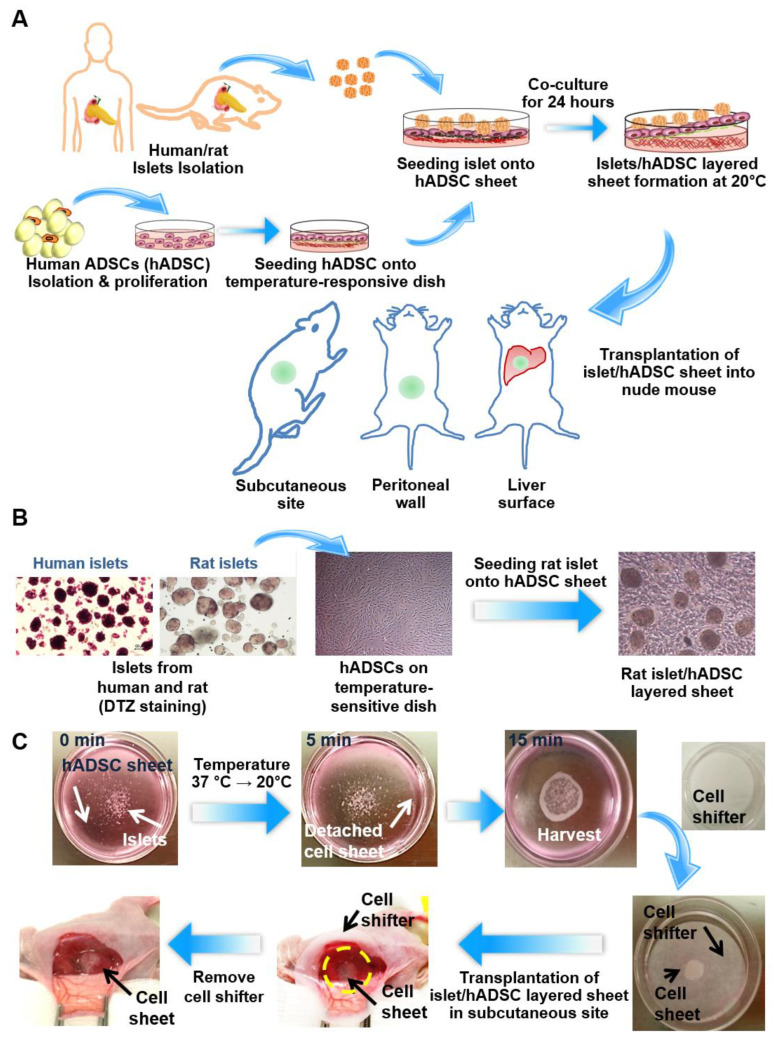
Multi-layered islet/adipose-derived stem cell (ADSC) sheet co-culture model and transplantation in three different sites. (**A**) Schematic diagram of layered Islet/ADSC (AI) sheet co-culture. Islets were isolated from humans and rats. Human ADSCs (hADSCs) were isolated from human fat tissue and cultured on temperature-response dishes to produce cell sheets. Isolated islets were seeded on ADSC sheets after the hADSCs reached confluence and were harvested after layered co-culture for 24 h. AI sheets were transplanted in the subcutaneous site, peritoneal wall, and liver surface. (**B**) Microscope image of isolated islets, an hADSC sheet, and an AI multi-layered sheet. (**C**) Experimental procedure of layered AI sheet co-culture and transplantation into the subcutaneous site.

Figure 1A shows the scheme for comparing cell sheet efficacy according to the transplantation sites in rats. Three different extrahepatic sites were used: the subcutaneous site, liver surface, and peritoneal wall. Figure 1B shows microscopy images of islets, ADSCs, and a multilayered islet/ADSC sheet. Human ADSCs were isolated and proliferated in vitro. The characteristics of isolated ADSCs were summarized in supplementary Figure S1. At passage 4, ADSCs were seeded on temperature-sensitive culture dishes and cultured until confluent for 2–3 days. Human and rat islets were isolated from the pancreas and seeded on UpCell^TM^ dishes after the ADSCs reached confluence. Islets exhibited good attachment on confluent ADSCs and detachment after the temperature was reduced to 20 °C (Figure 1C). After detachment, the cell sheet was folded in half, which encapsulated the islets, and transplanted using a Cellshifter^TM^ membrane.

### 3.2. Islet/ADSC (AI) Sheet Groups Showed Better Viability and Glucose Response Than the Islet-Only Group during the Culture Period

**Figure 2:**
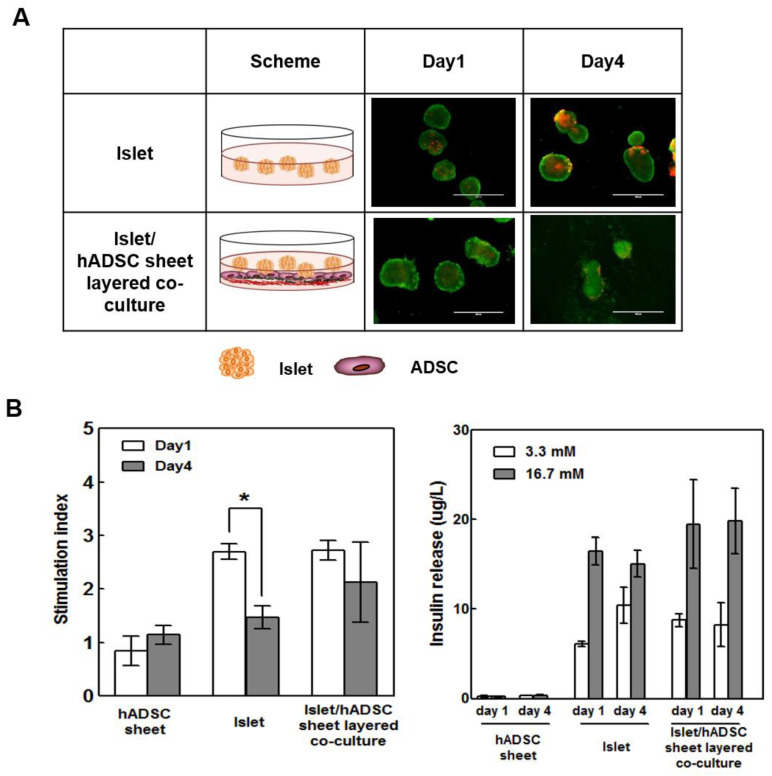
AI sheet groups showed better viability and glucose response than the islet-only group during the culture period. (**A**) The viability of islets and AI sheets was assessed by LIVE/DEAD staining. (**B**) Insulin release at 3.3 mM and 16.7 mM glucose in the hADSC sheet, islets, and AI sheet (in triplicate). The glucose-stimulated insulin secretion (GSIS) test showed better glucose response by the AI sheet group than by the islet-only group on days 1 and 4. Hundred hand-picked rat islets were used for each well. Results are presented as mean ± SD. Statistical significance was determined by a t-test of the stimulation index on days 1 and 4; * *p* < 0.05, *n* = 5.

We compared the viability and function of islets cultured in multi-layered sheets with that of the islet-only group during the culture period. AI sheets showed better viability than islets alone in the LIVE/DEAD viability/cytotoxicity assay (Figure 2A). Furthermore, AI multi-layered sheets showed better glucose response than islets alone, with a better glucose-stimulated insulin secretion (GSIS) index at days 1 and 4 of culture (Figure 2B). On day 1, both islet and islet/sheet groups showed similar glucose-sensitive responses, but the islet-only group exhibited significantly decreased glucose response after culture. These results indicate that ADSCs can play a cytoprotective role in islet cell survival after culture or transplantation, and AI sheets can be implemented as effective tools for transplantation.

### 3.3. Subcutaneous AI Sheet Transplantation Showed Better Blood Glucose Control Than Islet-Only Transplantation in Diabetic Nude Mice

**Figure 3:**
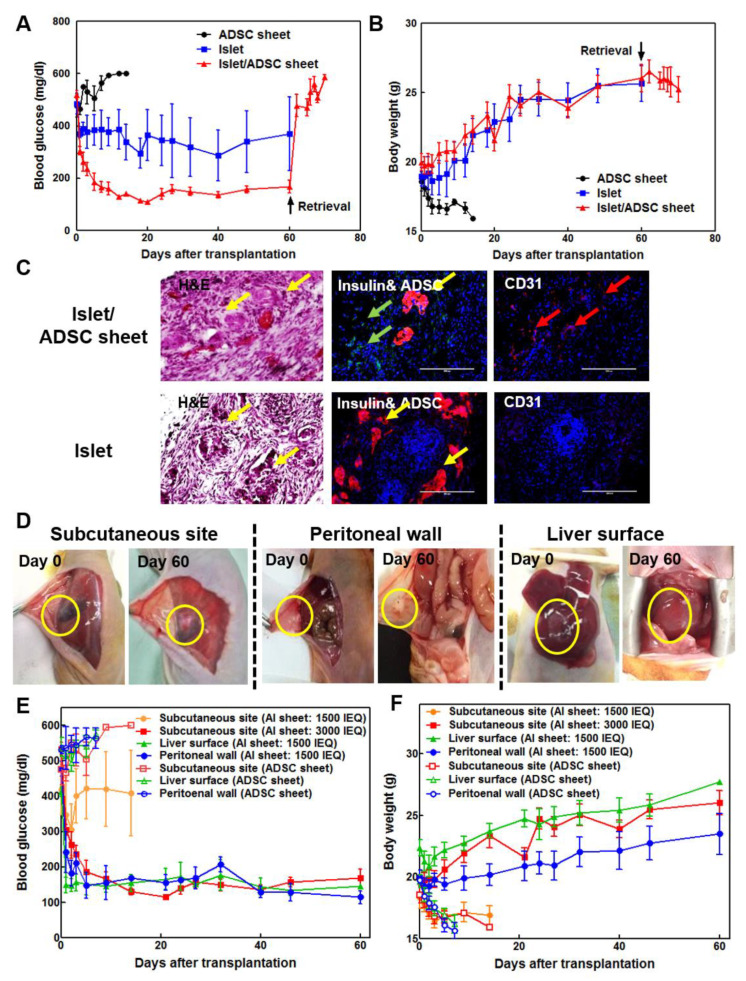
(**A**) Subcutaneous transplantation of rat islets with ADSC sheets showed better blood glucose control than transplantation of islets alone in diabetic nude mice (*n* = 5). Rat islets (3000 IEQ) with ADSC sheet showed more favorable blood glucose levels than islets alone. (**B**) The body weight of the islet-only and AI sheet groups was increased compared to that of the ADSC control group. Islet and AI sheet group showed significantly lower blood glucose levels and higher body weight (*p* < 0.05). (**C**) Hematoxylin-eosin (H&E), insulin, and CD31 staining of tissues from mice transplanted with AI sheet and islets only. In the AI sheet group, ADSCs adequately surrounded the transplanted islets and induced angiogenesis (upper panel) compared to islet-only transplants (lower panel). Yellow arrow: islets, green arrow: ADSCs, red arrow: vessels. Scale bar: 200 µm. (**D**) Transplantation of AI sheet on the subcutaneous site (*n* = 5), peritoneal wall (*n* = 4), and liver surface (*n* = 5) was performed successfully. (**E**,**F**) Blood glucose levels and body weights after transplanting ADSC sheet on the subcutaneous site (3000 IEQ and 1500 IEQ), liver surface (1500 IEQ), and peritoneal wall (1500 IEQ). Mouse transplanted with ADSC sheet without islets at each transplantation sites are sham operation control (*n* = 3). Mouse transplanted with 1500 IEQ AI sheet showed high blood glucose level and weight loss, indicating that 1500 IEQ islet is not enough to control diabetes at subcutaneous site. However, 1500 IEQ AI sheet transplanted on liver surface or peritoneal wall could reduce blood glucose level to that of normal glycemia. The 3000 IEQ AI sheet also showed normal glycemia. Body weight of subcutaneous site (AI sheet: 3000 IEQ) and liver surface (AI sheet: 1500 IEQ) groups is statistically higher than that of subcutaneous site (AI sheet: 1500 IEQ), peritoneal wall (AI sheet: 1500 IEQ), and sham operation (ADSC sheet) groups. Blood glucose level of subcutaneous site (AI sheet: 3000 IEQ), liver surface (AI sheet: 1500 IEQ), and peritoneal wall (AI sheet: 1500 IEQ) groups is statistically higher than that of subcutaneous site (AI sheet: 1500 IEQ) and sham operation (ADSC sheet) groups. (*p* < 0.05).

AI sheets were transplanted into diabetic nude mice subcutaneously at 3000 islet equivalents (IEQ). An equal number of islets or ADSC sheets were transplanted separately as controls. AI sheets showed superior glucose control in diabetic nude mice compared to control groups, which decreased below 200 mg/dL throughout the transplantation period and increased dramatically after graft retrieval (Figure 3A). Islet-transplanted mice showed slightly, but not completely, decreased blood glucose levels. Body weight was increased in both AI sheet and islet transplantation groups, although blood glucose was not normalized in the latter group (Figure 3B). The ADSC sheet-only group showed severe diabetes, and all animals were euthanized within 2 weeks due to sharply decreased body weight. Intact islets and insulin can be identified in grafts with H&E and immunofluorescent staining in both AI and islet-only transplantation. However, the vascular marker CD31 was more abundant with AI than with islets alone (Figure 3C).

### 3.4. Optimization of Transplantation sites of Rat AI Sheets

Because of the adhesive properties of cell sheets, AI sheets can be effectively transplanted on various organ surfaces. Particularly, cell sheets on the liver surface or peritoneal wall may show poor adherence or easy detachment due to an intact epithelial surface; additionally, the transplantation site is exposed to the abdominal cavity, unlike the subcutaneous site. To solve this problem, a rough surface was generated by scratching with dry gauze before transplantation. The transplantation sites showed good cell sheet attachment through the 2-month period and numerous blood vessels (Figure 3D). Preliminary experimentation confirmed that peritoneal walls or liver surfaces were richer in blood vessels than subcutaneous sites, and blood glucose levels were controlled even with less than half the number of transplanted cells. For verification, 1500 islets with the ADSC sheet were transplanted in the subcutaneous site, liver surface, and peritoneal wall. In the subcutaneous site, blood glucose was not regulated after decreasing the islet number from 3000 to 1500, but the liver surface and peritoneal wall maintained normal glucose levels throughout the transplantation period with 1500 IEQ (Figure 3E,F). All mice transplanted with 1500 IEQ AI sheet in subcutaneous site were euthanized at 2 weeks after transplantation because of severe weight loss of diabetics. Moreover, ADSC sheets alone were transplanted in subcutaneous site, on liver surface or peritoneal wall of diabetic mice. When only ADSC sheet was transplanted, no recovery of diabetes was observed in any group, and weight loss due to diabetes was observed. The control group was euthanized according to animal ethics due to rapid weight loss caused by diabetes within 1 week (liver surface and peritoneal wall groups) and 2 weeks (subcutaneous site group) after transplantation.

### 3.5. Comparison of Human AI Sheet Transplantation Among the Subcutaneous Site, Liver Surface, and Peritoneal Wall

**Figure 4:**
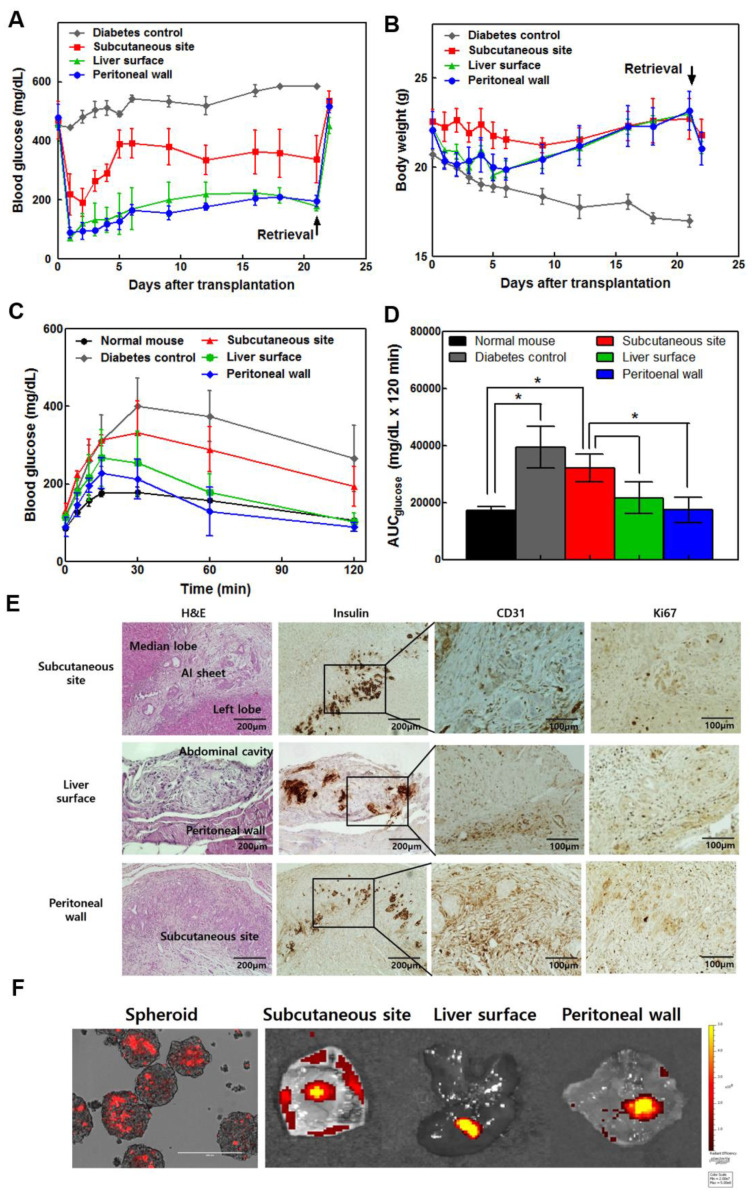
The liver and peritoneal surface were more effective sites for cell sheet transplantation among subcutaneous, liver surface, and peritoneal wall sites using human islets (5000 IEQ) and ADSC multi-layered sheets in diabetic nude mice (*n* = 4). (**A**) Transplantation of AI sheets onto the three sites was performed successfully. The liver and peritoneal surface were more effective for controlling blood glucose levels. (**B**) The liver surface and peritoneal wall groups showed increased body weight. The subcutaneous group-maintained body weight, but diabetic control mice dramatically decreased in weight. (**C**) Intraperitoneal glucose tolerance test (IPGTT). The mice were fasted for 8 h, and then 2 g glucose/kg body weight was injected intraperitoneally. The baseline blood glucose was measured at 0, 15, 30, 60, and 120 min. The blood glucose profile (**C**) and area under the curve (AUC) (**D**) during IPGTT in the peritoneal wall were similar to those of non-diabetic mice. The liver surface and peritoneal wall groups showed significantly controllable results compared to the subcutaneous site group. Values are expressed as means ± SD. (* *p* < 0.05). (**E**) H&E and immunohistochemical staining for insulin, CD31, and Ki67 at 3 weeks after transplantation of AI sheets in three different sites. (**F**) PKH26 red fluorescent cell linker-labeled insulinoma spheroid with ADSC sheet was examined after transplantation with an in vivo imaging system.

To investigate the effect of the location of AI sheet transplantation, we isolated human islets from a discarded pancreas specimen after pancreatic surgery, which was approved by the IRB of Asan Medical Center. We isolated human islets from discarded human pancreas tissue resected due to benign pancreatic disease after obtaining the informed consent from patients. Human islet preparations were analyzed for purity, glucose stimulated insulin secretion, and in vivo transplantation, these data was presented in supplementary Figure S2. We performed AI cell sheet transplantation onto the subcutaneous site, liver surface, and abdominal peritoneal wall (Figure 4A,B). Blood glucose and weight gain were more effectively controlled in mice bearing AI sheets on the liver surface or peritoneal wall than in the subcutaneous site. Blood glucose was elevated after graft removal. Although mice with AI sheet transplantation on both the liver surface and peritoneal wall showed increased body weight during the transplantation period, the weight initially decreased sharply on day 1 due to open-abdominal surgery. Diabetic control mice showed high blood glucose levels and severely decreased body weight, but subcutaneous-transplanted mice-maintained body weight without an initial reduction due to surgery. Mice with AI sheet transplantation on the liver surface or peritoneal wall also showed better IPGTT profiles similar to normal non-diabetic mice (Figure 4C,D). Peritoneal wall-transplanted mice showed AUCs lower than other diabetic and transplanted groups, similar to that of normal mice. Histology confirmed intact islets and insulin-producing cells in mouse grafts (Figure 4E). Abundant neovascularization was observed at the transplanted site on liver surface, peritoneal wall, and subcutaneous site, but there was no significant difference among the three groups. In addition, we conducted immunohistochemical staining for Ki67 to assess islet replication in transplanted sites. The proliferating islets were confirmed to be insulin/Ki67 double-positive cells. Ki67 positive cells were identified around the islets, but few cells proliferated around the islets. We used adult islets in the study, which rarely proliferate after transplantation. To confirm survival after cell transplantation in various sites, we prepared rat islet insulinoma cell clusters labeled with PKH26. Mice were sacrificed and non-invasive fluorescence images were obtained (Figure 4F). The spheroid made of insulinoma labeled with PKH26 formed 200 µm-sized spheroids similar to islets and exhibited red fluorescence. One week after the AI sheet was implanted, each implantation site was collected, and fluorescence was confirmed ex vivo using IVIS to confirm the distribution of the transplanted cells. Fluorescence was observed in all of the subcutaneous, liver surface, and peritoneal wall sites where AI sheet was implanted. Higher fluorescence signals were observed in the liver and peritoneal wall than in the subcutaneous site.

## 4. Discussion

Islet transplantation has proven to be an effective treatment in T1D, but many obstacles remain before it can be broadly applied. Clinically, pancreatic islet transplantation is conducted via the intraportal route [28]. The need for numerous islets to achieve euglycemia and progressive deterioration in islet function over time represents significant barriers [4,29]. Islets injection through portal vein may contribute to islet dysfunction and loss [30]. Thus, the development of alternative islet transplantation sites is a priority. The renal subcapsular space is a well-established site for islet transplantation in rodent models, but anatomical differences have precluded the use of this site in nonhuman primates and humans. The subcutaneous space has been suggested as a good candidate site for islet transplantation [31,32]. Although the subcutaneous site is safe and minimally invasive, it shows insufficient blood supply. Therefore, organs with sufficient blood supply, such as the liver and peritoneum, are good alternatives. Our study demonstrates the characteristics of each islet implantation site using cell sheet technology, including the subcutaneous site, liver surface, and peritoneal wall, and the advantages of each regarding islet engraftment and post-transplant function.

However, in practice, it is difficult to transplant cells on organs without a pocket for cell transplantation on the liver surface or peritoneal wall that confines them to the target site using a general injection method. Because cell sheets produced on temperature-sensitive dishes have adhesion proteins attached to the underside, they can be easily attached to various organs, allowing transplantation. Cell sheet techniques, in which cultured cells are harvested as intact sheets, along with their deposited extracellular matrix (ECM), were developed using temperature-responsive surfaces, allowing direct transplantation or overlapping three-dimensional tissue-like structures. Additionally, when the tissues are damaged, the injected cells may be unable to attach at sites where the host architecture is destroyed. By contrast, cell sheets with deposited ECM can attach to host tissues and wound sites, covering the surface with minimal cell loss. Previously, the Okano group produced and transplanted a cell sheet using single cell of islets to control diabetes, but the islets reverted to the native form, which limited its clinical application [19,20,21].

Recently, study of manufacturing sheets containing cells with supportive roles, such as MSCs and fibroblasts, while maintaining islet structure and their transplantation into composites has shown good results [18,22]. Cell sheets comprising islets and supporting cells have shown beneficial effects for islet survival and function, including the maintenance of islet morphology and preservation of ECM components. Particularly, MSCs can serve as an effective support because they aid islet survival and function through various growth factors and regulate the immune response after transplantation. Several studies have described the usefulness of islet transplantation using MSCs [33,34,35], but islet transplantation using cell sheet-shape of MSCs is considered more useful because of its easy and practical approach. We verified that viability and physiological function of islets increased in a sheet composite group, which was consistent with a previous study [22]. These results indicated that ADSCs were associated with higher islet viability and function.

Because islet cell sheet technology for subcutaneous site transplantation is regarded as the safest, simplest, and least invasive method, we investigated engraftment efficacy by transplanting islet alone or AI sheets on the subcutaneous tissue of diabetic athymic mice. Our results showed that ADSC sheet effectively supported islet engraftment and function in vivo. In contrast, islet survival was not observed in any subcutaneous transplants of islets or ADSC sheets separately, suggesting that AI sheets improve the islet engraftment rate at subcutaneous sites. However, a large mass of islets (3000 IEQ rat islets per mouse) was required to achieve euglycemia in subcutaneous site of diabetic mice. The major reason for this insufficiency was that transplanted islets were not viable and functional due to limited oxygen and nutritional supplies caused by the lack of early neovascularization in the subcutaneous site [23]. To identify the optimal islet transplantation site, we compared the liver surface, peritoneal wall, and subcutaneous site as transplantation sites. The peritoneal wall and liver surface groups showed more consistent glycemic control than the subcutaneous site group. We found that relatively few cells (1500 IEQ) were needed to provide persistent normoglycemia in the peritoneal wall and liver surface groups. When the subcutaneous site was used for islet transplantation, twice as many islets were required to restore normoglycemia in diabetic mice. In the case of transplantation to the liver and peritoneal wall, there was an advantage of reducing the number of islets to be transplanted, but there was weight loss immediately after surgery. In this study, rapid weight loss was observed in mouse transplanted with rat AI sheet on the peritoneal wall. The weight of animals implanted in the peritoneal wall was lower than that of animals implanted in the liver surface or subcutaneously. When rat islet/ADSC sheets were transplanted into the peritoneal wall, body weight decreased in peritoneal wall group compared to that in liver surface group. However, when human islet/ADSC sheets were transplanted onto liver surface or peritoneal wall, body weight decreased immediately after surgery but tended to recover quickly in both groups. Usually, transplanting islets to diabetic mouse prevents weight loss in diabetes and leads to an increase in body weight. In addition, loss of weight by surgery causes the transplanted islets to gradually increase weight gain; this weight gain is considered to differ in animals with different conditions depending on the degree of function of the transplanted cells. In animals implanted in the peritoneal wall, we thought that the human islet function was better than the rat islet function. However, it is necessary to further study whether it is due to species differences or the amount of transplanted cells, because the number of available studies on optimization of the exact number of transplanted islets is insufficient.

To confirm these results, we isolated human islets from partial pancreatectomy tissues and transplanted human islets/ADSC sheets. Previously, we usually transplanted rat islets at 500–1000 IEQ into the kidney capsule for glucose control, but human islets were transplanted at 2000–3000 IEQ. Based on the kidney capsule transplant, we transplanted 5000 IEQ human islets, as estimated by proportional calculations. Furthermore, transplantation of an optimized human islet preparation with viable and functionally potent islets is a key prerequisite for successful transplantation regardless of other transplantation conditions. To assess the function of islets isolated from surgical specimen, we conducted GSIS assay and in vivo islet transplantation in kidney capsule of diabetic mice.

To assess the efficiency of cell sheets with human islets, we transplanted human islets and ADSC sheets in three different sites. Similar to the findings from rodent islet transplantation experiments, blood glucose levels were immediately normalized in the peritoneal wall and liver surface, but not the subcutaneous site. Although circulating insulin was not measured, graftectomy was performed and all mice became hyperglycemic, indicating that blood glucose regulation was strictly due to the transplanted cell sheet. These results suggest that circulating insulin contributes to blood glucose regulation. Particularly, the peritoneal wall and liver surface groups showed stable blood glucose levels and produced a glycemic AUC that was most similar to that of normal animals in terms of the IPGTT, reflecting physiologically normal insulin delivery. These results suggest that the intraperitoneal wall and liver surface are favorable transplantation sites for AI sheets.

The peritoneum, a serous membrane that lines the abdominal cavity walls, forms a natural biological membrane where micro-vessels are distributed in the peritoneal tissues. Notably, a portion of the blood circulation of the peritoneum leads directly into the portal venous system. Hence, any insulin absorbed by the peritoneum would potentially have nearly direct access to the liver. Consequently, such insulin would first be available to reduce hepatic glucose production and could, therefore, potentially regulate glucose more effectively. Additionally, the liver surface was recently reported as an alternative transplantation site by avoiding the risks posed by the portal vein. Our results also showed that the peritoneal wall and liver surface were efficacious in reversing diabetes post-transplant in mice at a marginal islet dose and had the advantage of higher vascular access compared to the subcutaneous site. In conclusion, we suggest that the peritoneal wall and liver surface should be considered as alternative implant sites and that minimal or suboptimal islet numbers may be sufficient to prevent hyperglycemia in the recipient.

## 5. Conclusions

This study aimed to improve islet transplantation efficiency and survival using ADSC sheets. Cell sheet technology and ADSCs can serve as useful tools for improving the outcome of islet cell transplantation since both can increase implantation efficacy and graft survival. A multi-layer sheet was produced by attaching native islets clusters to stem cell-sheet. We optimized the transplanted sites through simultaneous transplantation to three different sites by using the attachment characteristics of the cell sheet. Firstly, the efficiency of subcutaneous islet transplantation of stem cell sheets was verified, and then the blood-rich and clinically usable sites of the liver surface and peritoneal wall were compared with the subcutaneous site. To increase the possibility of clinical application, this finding was verified by applying human islets, as well as rat islets. Therefore, the liver and peritoneal surface can be used more effectively for AI sheet transplantation than the subcutaneous site in future clinical applications.

## Data Availability

The data that support the findings of this study are available on request from the corresponding authors.

## Supplementary Materials

The following are available online at https://www.mdpi.com/2073-4409/9/9/1999/s1. Figure S1: Characteristic of isolated hADSCs; Figure S2: Quality control for human islet isolation and transplantation.

## References

[1] Ichii H., Inverardi L., Pileggi A., Molano R.D., Cabrera O., Caicedo A., Messinger S., Kuroda Y., Berggren P.-O., Ricordi C. (2005). A novel method for the assessment of cellular composition and beta-cell viability in human islet preparations. Am. J. Transplant..

[2] Alejandro R., Latif Z., Noel J., Shienvold F.L., Mintz D.H. (1987). Effect of anti-Ia antibodies, culture, and cyclosporin on prolongation of canine islet allograft survival. Diabetes.

[3] Contreras J.L., Eckstein C., Smyth C.A., Bilbao G., Vilatoba M., Ringland S.E., Young C., Thompson J.A., Fernández J.A., Griffin J.H. (2004). Activated protein C preserves functional islet mass after intraportal transplantation: A novel link between endothelial cell activation, thrombosis, inflammation, and islet cell death. Diabetes.

[4] Ryan E.A., Paty B.W., Senior P.A., Bigam D., Alfadhli E., Kneteman N.M., Lakey J.R.T., Shapiro A.M.J. (2005). Five-year follow-up after clinical islet transplantation. Diabetes.

[5] van der Windt D.J., Bottino R., Casu A., Campanile N., Cooper D.K.C. (2007). Rapid loss of intraportally transplanted islets: An overview of pathophysiology and preventive strategies. Xenotransplantation.

[6] Korsgren O., Lundgren T., Felldin M., Foss A., Isaksson B., Permert J., Persson N.H., Rafael E., Rydén M., Salmela K. (2008). Optimising islet engraftment is critical for successful clinical islet transplantation. Diabetologia.

[7] Merani S., Toso C., Emamaullee J., Shapiro A.M.J. (2008). Optimal implantation site for pancreatic islet transplantation. Br. J. Surg.

[8] Carlsson P.O., Palm F., Andersson A., Liss P. (2000). Chronically decreased oxygen tension in rat pancreatic islets transplanted under the kidney capsule. Transplantation.

[9] Fritschy W.M., van Straaten J.F., de Vos P., Strubbe J.H., Wolters G.H., van Schilfgaarde R. (1991). The efficacy of intraperitoneal pancreatic islet isografts in the reversal of diabetes in rats. Transplantation.

[10] Ao Z., Matayoshi K., Lakey J.R., Rajotte R.V., Warnock G.L. (1993). Survival and function of purified islets in the omental pouch site of outbred dogs. Transplantation.

[11] Kaufman D.B., Morel P., Field M.J., Munn S.R., Sutherland D.E. (1990). Purified canine islet autografts. Functional outcome as influenced by islet number and implantation site. Transplantation.

[12] Cantarelli E., Piemonti L. (2011). Alternative transplantation sites for pancreatic islet grafts. Curr. Diabetes Rep..

[13] Kobayashi J., Kikuchi A., Aoyagi T., Okano T. (2019). Cell sheet tissue engineering: Cell sheet preparation, harvesting/manipulation, and transplantation. J. Biomed. Mater. Res..

[14] Owaki T., Shimizu T., Yamato M., Okano T. (2014). Cell sheet engineering for regenerative medicine: Current challenges and strategies. Biotechnol. J..

[15] Fujita I., Utoh R., Yamamoto M., Okano T., Yamato M. (2018). The liver surface as a favorable site for islet cell sheet transplantation in type 1 diabetes model mice. Regen. Ther..

[16] Hopcroft D.W., Mason D.R., Scott R.S. (1985). Structure-function relationships in pancreatic islets: Support for intraislet modulation of insulin secretion. Endocrinology.

[17] Lammert E., Thorn P. (2020). The Role of the Islet Niche on Beta Cell Structure and Function. J. Mol. Biol..

[18] Hirabaru M., Kuroki T., Adachi T., Kitasato A., Ono S., Tanaka T., Matsushima H., Sakai Y., Soyama A., Hidaka M. (2015). A Method for Performing Islet Transplantation Using Tissue-Engineered Sheets of Islets and Mesenchymal Stem Cells. Tissue Eng. Part. C Methods.

[19] Shimizu H., Ohashi K., Utoh R., Ise K., Gotoh M., Yamato M., Okano T. (2009). Bioengineering of a functional sheet of islet cells for the treatment of diabetes mellitus. Biomaterials.

[20] Saito T., Ohashi K., Utoh R., Shimizu H., Ise K., Suzuki H., Yamato M., Okano T., Gotoh M. (2011). Reversal of diabetes by the creation of neo-islet tissues into a subcutaneous site using islet cell sheets. Transplantation.

[21] Shimizu H., Ohashi K., Saito T., Utoh R., Ise K., Yamato M., Okano T., Gotoh M. (2013). Topographical arrangement of α- and β-cells within neo-islet tissues engineered by islet cell sheet transplantation in mice. Transplant. Proc..

[22] Imamura H., Adachi T., Kin T., Ono S., Sakai Y., Adachi T., Soyama A., Hidaka M., Takatsuki M., Shapiro A.M.J. (2018). An engineered cell sheet composed of human islets and human fibroblast, bone marrow-derived mesenchymal stem cells, or adipose-derived mesenchymal stem cells: An in vitro comparison study. Islets.

[23] Sakata N., Aoki T., Yoshimatsu G., Tsuchiya H., Hata T., Katayose Y., Egawa S., Unno M. (2014). Strategy for clinical setting in intramuscular and subcutaneous islet transplantation. Diabetes Metab. Res. Rev..

[24] Wang W., Gu Y., Tabata Y., Miyamoto M., Hori H., Nagata N., Touma M., Balamurugan A.N., Kawakami Y., Nozawa M. (2002). Reversal of diabetes in mice by xenotransplantation of a bioartificial pancreas in a prevascularized subcutaneous site. Transplantation.

[25] Kim J.-S., Lim J.-H., Nam H.-Y., Lim H.-J., Shin J.-S., Shin J.-Y., Ryu J.-H., Kim K., Kwon I.-C., Jin S.-M. (2012). In situ application of hydrogel-type fibrin-islet composite optimized for rapid glycemic control by subcutaneous xenogeneic porcine islet transplantation. J. Control. Release.

[26] Ricordi C., Lacy P.E., Finke E.H., Olack B.J., Scharp D.W. (1988). Automated method for isolation of human pancreatic islets. Diabetes.

[27] Jeong J.J., Lee D.W., Song S.Y., Park Y., Kim J.H., Kim J.I., Kim H.G., Nam K.T., Lee W.J., Nam K.-H. (2019). Development of novel biocompatible thermosensitive anti-adhesive agents using human-derived acellular dermal matrix. PLoS ONE.

[28] Shapiro A.M., Pokrywczynska M., Ricordi C. (2017). Clinical pancreatic islet transplantation. Nat. Rev. Endocrinol..

[29] Matsumoto S. (2010). Islet cell transplantation for Type 1 diabetes. J. Diabetes.

[30] Li X., Meng Q., Zhang L. (2018). The Fate of Allogeneic Pancreatic Islets following Intraportal Transplantation: Challenges and Solutions. J. Immunol. Res..

[31] Fukuda S., Yabe S.G., Nishida J., Takeda F., Nashiro K., Okochi H. (2019). The intraperitoneal space is more favorable than the subcutaneous one for transplanting alginate fiber containing iPS-derived islet-like cells. Regen. Ther..

[32] Pathak S., Regmi S., Gupta B., Pham T.T., Yong C.S., Kim J.O., Yook S., Kim J.-R., Park M.H., Bae Y.K. (2017). Engineered islet cell clusters transplanted into subcutaneous space are superior to pancreatic islets in diabetes. FASEB J..

[33] Paek H.J., Kim C., Williams S.K. (2014). Adipose stem cell-based regenerative medicine for reversal of diabetic hyperglycemia. World J. Diabetes.

[34] Ohmura Y., Tanemura M., Kawaguchi N., Machida T., Tanida T., Deguchi T., Wada H., Kobayashi S., Marubashi S., Eguchi H. (2010). Combined transplantation of pancreatic islets and adipose tissue-derived stem cells enhances the survival and insulin function of islet grafts in diabetic mice. Transplantation.

[35] Bhang S.H., Jung M.J., Shin J.-Y., La W.-G., Hwang Y.H., Kim M.J., Kim B.-S., Lee D.Y. (2013). Mutual effect of subcutaneously transplanted human adipose-derived stem cells and pancreatic islets within fibrin gel. Biomaterials.

