# Supplementary material for: Evaluation of Multi-Layered Pancreatic Islets and Adipose-Derived Stem Cell Sheets Transplanted on Various Sites for Diabetes Treatment
Source: Cells. 2020 Aug 31;9(9):1999. doi: 10.3390/cells9091999 (PMC7563383; doi:10.3390/cells9091999)
Supplement: Supplementary file 1 [file cells-09-01999-s001.pdf]

Evaluation of multi-layered pancreatic islets and adipose-derived stem cell sheets transplanted on various sites for diabetes treatment

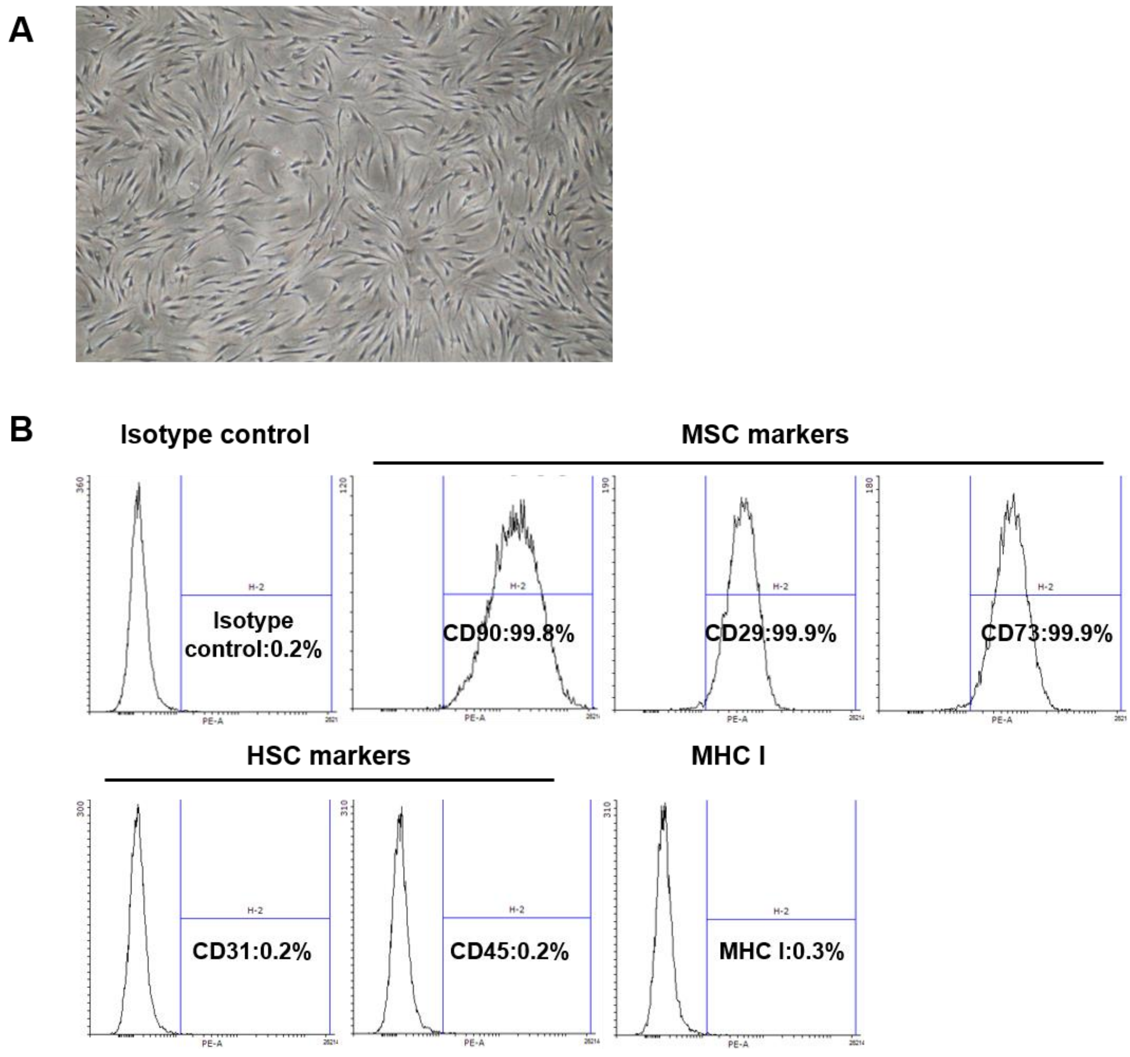

Figure S1. Characteristics of isolated hADSCs. (A) Morphology of ADSCs and (B) Flow cytometry analysis of surface markers expression including CD90, CD29, CD73, CD31, CD45 and MHC I in ADSCs at passage 3 of *in vitro* culture.

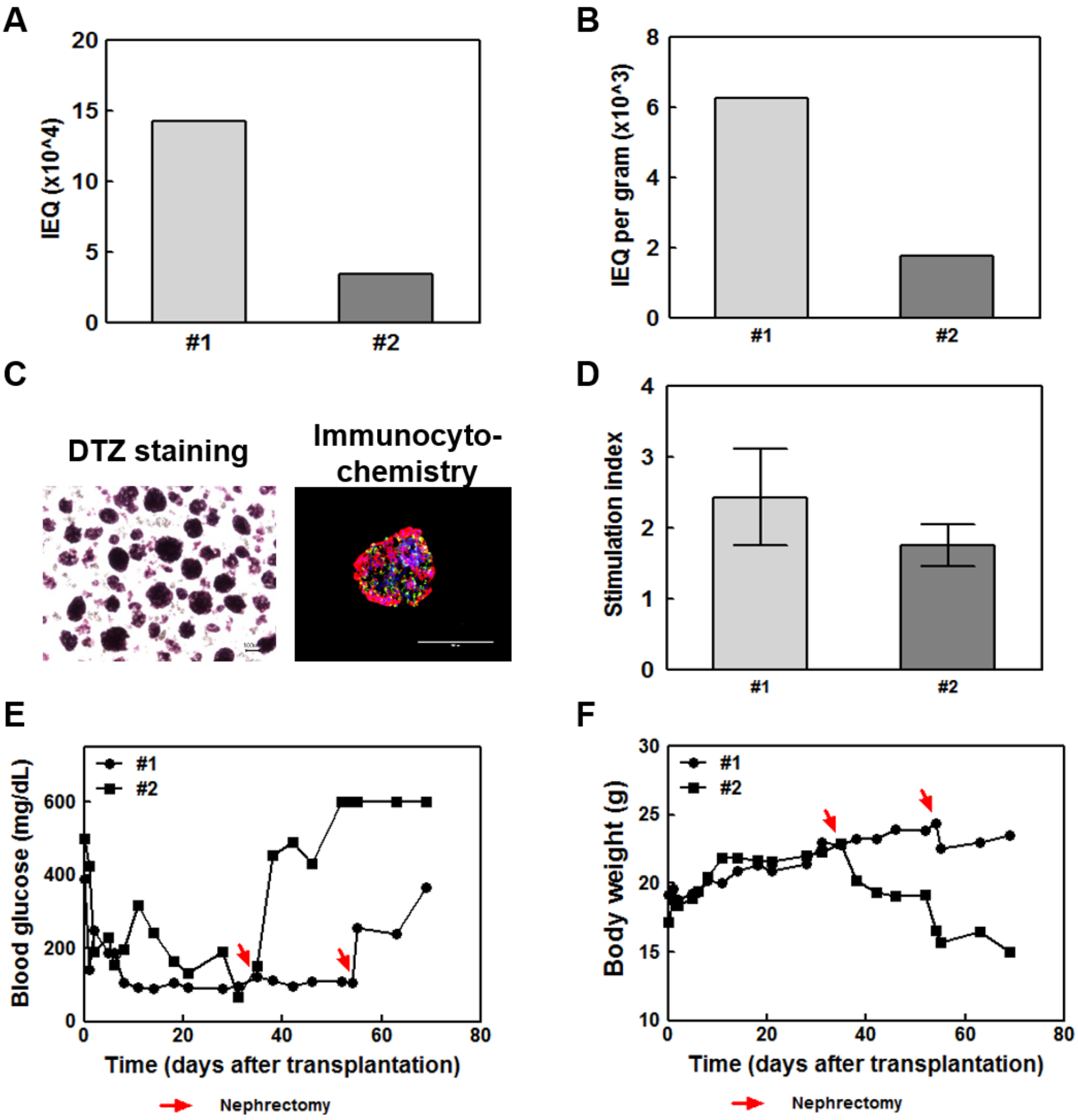

Figure S2. Quality control for human islet isolation and transplantation. (A) Islet yield. (B) Islet equivalents (IEQ) per gram of pancreatic tissue digested. (C) Islets were stained with dithizone (left, scale bar = 200 μm) and immunostained with antibodies against insulin (green) and glucagon (red). Alpha and beta cells showed regular and normal distribution (right). (D) GSIS in human islets. For in vivo quality control of isolated human islets, 2000 IEQ islets were transplanted under the kidney capsule of diabetic mice, and blood glucose levels (E) and body weight (F) were measured. Kidneys containing grafts were removed on days 35 and 54 post-transplantation (arrows).
